# An integrative review of the characteristics of meaningful learning in healthcare professionals to enlighten educational practices in health care

**DOI:** 10.1002/nop2.3

**Published:** 2014-10-01

**Authors:** Lucia Cadorin, Annamaria Bagnasco, Gennaro Rocco, Loredana Sasso

**Affiliations:** ^1^ Department of Health SciencesUniversity of GenoaI‐16132GenoaItaly; ^2^ Centre of Excellence for Nursing Scholarship78 I‐00192RomeItaly

**Keywords:** Constructivist paradigm, healthcare professionals, integrative review, meaningful learning, nurse, significant learning

## Abstract

**Aim:**

Knowledge does not transfer automatically, but requires an active, personal progress through meaningful learning. As posited by the constructivist paradigm, the aim of this study was to identify the characteristics of meaningful learning by analysing definitions and correlated methods found in the literature.

**Design:**

An integrative review.

**Methods:**

Articles were sought on MEDLINE, CINAHL and SCOPUS; no language, time or study‐design restrictions were adopted. Only papers referring explicitly to the diverse types of learning were taken into account; 11 articles were included in this review.

**Results:**

Findings from the literature revealed three different types of meaningful learning: (1) meaningful learning as ‘active building‐up process’; (2) meaningful learning as ‘change’; 3‐meaningful learning as ‘outcome of experience’. A focus on constructivism and meaningful learning provides a new outlook on healthcare professionals in learning, including nurses, who are gradually taking on greater responsibility in self‐ and ongoing education.

## Introduction

In this paper, we investigate how meaningful learning is rooted into constructivism, a type of learning that learners themselves build up so that they change the meaning of their experience. In health professionals, meaningful learning helps them to learn in a more effective way, use their knowledge in a more productive manner and contribute to improve the quality of their practice. Therefore, it is important to know the characteristics of meaningful learning and its methods.

In healthcare, the term ‘learning’ is frequently used to define an experience‐based process generating long‐term behavioural change, as well as the acquisition of knowledge, abilities and competence. Learning has been defined as ‘a process whereby individuals assimilate and gradually elaborate on increasingly complex and abstract units of knowledge, such as concepts, categories, behavioural schemata or models and/or whereby behavioural skills and competences are mastered’ (De Sanctis 2006).

Unlike scientific theories, educational theories do not slot into clear‐cut definitions, as they are not necessarily the result of experimental manipulation of controlled variables through a hypothetical‐deductive approach. In fact, educational theories are often the result of observation and assessment of teaching and learning practice based on an inductive approach (Dennick 2012). In the course of time, such approaches have led to different perspectives, such as Behaviourism, Cognitivism and Constructivism – each supported by underlying paradigms. This, in turn, has led to the development of different learning theories. Major awareness of the characteristics and of the methods to develop and implement meaningful learning may enlighten future educational practices and curricula for healthcare professionals.

### Background

Nurse educators are currently rethinking traditional teacher‐centred curriculum designs with the aim of embracing new ideologies with a stronger focus on learner‐centred learning (Stanley & Dougherty 2010). This paradigm reflects recommendations issued in 2012 by the National League for Nursing as an attempt to address the ever‐changing needs of nurses. Moreover, it is also in keeping with recommendations by Benner *et al*. (2010) on re‐visiting nursing education, meeting present and future patient expectations and teaching trainees to ‘be’ nurses rather than simply ‘practice’ nursing (Benner *et al*. 2010, Benner 2012, National League for Nursing 2012).

From a preliminary literature search targeting definitions and correlated underlying methods of different learning‐types, we focused our attention on identifying the characteristics of meaningful learning as posited by the constructivist paradigm. We selected constructivism as a reference model, as it allows for a specific person‐centred focus on individuals in training, which is in line with the characteristics displayed by healthcare students. Considered holistically, individuals are, therefore, viewed as part of a living environment wherein they play an active learning role. Meaningful learning clearly emerges from the constructivist paradigm. Ausubel *et al*. (1978) used the term ‘meaningful’ to describe the interaction between newly acquired and existing information (Ausubel *et al*. 1978). Instead, mechanical learning occurs when new information is acquired through memorization, with no integration with previously existing knowledge. This interaction constitutes the ‘knowledge building process’ that is one of the fundamental principles of constructivism.

#### The conceptual framework of meaningful learning

The conceptual framework of meaningful learning is based on constructivism, which is different from behaviourism and cognitivism. In behaviourism, knowledge is viewed as a passive, automatic response to external factors, or a reaction to specific environmental stimuli. Therefore, learning is only viewed in terms of behavioural change. Cognitivism, on the other hand, ignores external factors, so that learning is construed solely as cognitive change (De Sanctis 2006). In the cognitive approach, knowledge is an abstract, symbolic representation of the individual mind (De Sanctis 2006).

Instead, constructivist learning is both active and personal (De Sanctis 2006, Giaconi 2008, Varisco 2011) – a structured process that originates from individual experience (Hrynchak & Batty 2012). This marks the shift from an objective, target content‐centred approach to a subjective, learner‐centred outlook.

In constructivism, learning is strongly conditioned by environmental stimuli such as culture, group dynamics, motivation and emotion (De Sanctis 2006). Therefore, from a constructivist perspective, the main pedagogical objective is not that of supplying individuals with chunks of new and diverse knowledge, but rather that of re‐working and transforming already‐acquired knowledge (Santoianni & Striano 2003).

## The study

### Design

An integrative review.

### Method

This study includes two phases:

1. A preliminary review of the various types of learning for healthcare professionals and then a focus on meaningful learning due to its strong connection with constructivism.
2. Identification of the characteristics of meaningful learning by analysing the definitions found in the literature and the methods related to it.

#### Search strategies

The literature focusing on different learning‐types was reviewed to identify characteristics of meaningful learning. Guidelines on integrative reviewing were drawn from Whittemore and Knafl (2005), as this method allows for a combination of diverse research methodologies used to develop theory and evidence‐based practice.

Articles were sought out on MEDLINE, CINAHL and SCOPUS, with the following keywords: meaningful learning, significant learning, transformative learning, vicarious learning, generative learning and reflective learning, nurse, nursing, health professional – in detail: nurses, nursing students, dental hygienist students and physiotherapy students, doctor of pharmacy, students of Health Information Management). Key words were derived from findings on the different types of learning (Table 1) and specific strategies were devised for each database. No language, time or study‐design restrictions were adopted. Papers whose titles and abstracts referred explicitly to diverse learning types were taken into account.

**Table 1:** Classification of learning according to the authors.

Learning types	Definitions and sources
Meaningful Learning Significant Learning	The process of interaction between new information acquired by the individual and the relevant knowledge structures he or she already possesses (Ausubel *et al*. 1978, Fink 2007)
Transformative Learning	Learning that promotes change and transformation (Parker & Myrich 2010)
Vicarious Learning	Learning through the experiences of another (Roberts 2010)
Reflective Learning	Learning through experiences and reflection (Liimatainen *et al*. 2001)
Generative Learning	Learning that incorporates existing knowledge with new ideas based on experimentation and open mindedness (Jonassen 1999, Cosentino 2002)

#### Screening of articles and selection criteria

**Figure 1:**
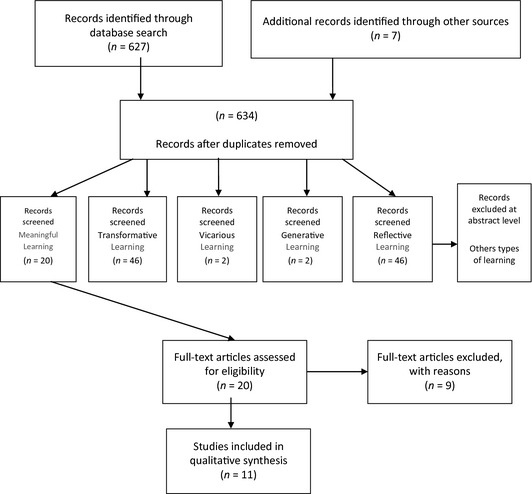
Flow diagram.

We retrieved 627 references with seven extra references found through searching other types of sources, for a total of 634 results. Duplicates and articles whose abstracts made unclear reference to learning types were ruled out. Papers were then streamed by learning type and data, including research number, author, title and journal, were entered into an *ad‐hoc* Excel table into which filters were then enabled; 11 articles are discussed in this review (Figure 1). All 11 papers were read and analysed by the authors individually. Definitions were used to draw up a list of the main concepts pertaining to meaningful learning, which were then shared and discussed. Prominent categories of characteristics pertaining to meaningful learning emerged from the discussion.

### Ethics

Ethical approval was not required.

## Results

Twenty papers dealt with meaningful learning, 46 dealt with transformative learning; two with vicarious learning; two with generative learning and 46 with reflective learning. This paper focuses on our integrative review relating only to the characteristics of meaningful learning, all other learning types will be discussed elsewhere (manuscript in preparation). Again, papers and authors with unclear references to constructivism were ruled out, so that a further nine papers were excluded.

This paper focuses on the information gleaned from the 11 selected papers. Table 2 reports the title, authorship, journal, place of implementation, conceptual references, study type and objectives, characteristics of meaningful learning and developmental methods for each examined paper. Where study design is concerned, eight papers referred to descriptive studies, one to a quasi‐experimental, pre‐post, exploratory study, one to a non‐randomized, pre‐post, controlled study and one to a non‐primary source, narrative review.

**Table 2:** Summary – review of the articles.

Author/year	Place	Conceptual reference	Type of study and objectives	Characteristics of meaningful learning	Methods to enhance meaningful learning
1‐Johannsen *et al*. (2012)	Dental hygienist (DH) students and physiotherapist (PT) students‐ Karolinska Institutet, Sweden	Three theories have been identified that are fundamental for students’ learning: constructivism, meaningful learning and self‐efficacy.	Study: descriptive Objectives: evaluating the implementation of the scholarship model and an evidence‐based approach to enhance meaningful learning and self‐efficacy amongst dental hygienist (DH) students and physiotherapist (PT) students.	‐Active process. ‐Deep and wide understanding.	The scholarship model was used that comprises the following steps: checking for the existence of knowledge; developing teaching practice; documenting the changes and the results of teaching; investigating what has happened thanks to the development of education; work‐hours and peer review; sharing of their work.
2‐Dreifuerst (2012)	Nursing students – U.S. University School of Nursing, USA	Definition of ‘meaningful learning’ according to the constructivist paradigm. Debriefing as a constructivist teaching strategy for meaningful learning (DML) is a method that uses guided reflection to encourage further development of clinical reasoning and thinking in nursing students.	Study: exploratory quasi‐experimental pre‐post Objectives: Describing and testing the relationship between debriefing for meaningful learning (DML) and the development of clinical reasoning skills of nursing students compared with normal debriefing.	‐Understanding of concepts. ‐Development of reasoning.	Use of debriefing for meaningful learning, a method for teaching clinical reasoning used in all types of simulation, including high‐fidelity simulation (HFS). The method uses a DML process, which involves six components: (a) undertake (the participants), (b) explore (Options reflection‐in‐action), (c) explain (decisions, actions and alternatives to deduction, induction and analysis), (d) process (thinking, broaden the analysis and inferential thinking), (e) evaluate (the experience of reflection in action) and (f) extend (deductive thinking and analytical reflection beyond action). Debriefing can develop meaningful learning and knowledge that facilitate clinical reasoning.
3‐Krueger *et al*. (2011)	University of Wyoming School of Pharmacy, USA PhD Pharmacy students, Nursing students and social work.	Fink's Taxonomy for meaningful learning.	Study: non‐randomized controlled, pre‐post. Primary objective: Evaluating whether a course redesign of health policies using Fink's taxonomy for meaningful learning increases students’ knowledge of health policies and health system and whether they are aware of the importance of this knowledge.	‐Lasting change	The following activities have been identified: ‐provided students with necessary reading to develop knowledge before class; ‐ Lecture on selected topics; ‐Simulation of activity; ‐ Health policy project to promote the application and integration of knowledge.
4‐Prado *et al*. (2011)	Nursing Degree, School of Nursing, University of São Paulo, San Paolo, Brazil	Significant learning according to the theoretical framework of Ausubel.	Study: descriptive Objectives: Developing and evaluating virtual classrooms according to the ‘theory of Meaningful learning’ available in Moodle.	‐Desire to learn. ‐Presence of relevant concepts to understand the new ones. ‐Logical and psychological meaning.	The project was developed in four stages: ‐ Design and planning; ‐ Content development and instructional design; ‐Design and creation of educational materials; ‐Creation of learning portal.
5‐Fink (2007)	Carolyn Fellahi, a Psychology Professor at Central Connecticut State University Professor of computer engineering at the University of Missouri–Rolla USA	Fink's Taxonomy for meaningful learning. According to Fink, it is represented by six types of learning: ‐Basic knowledge ‐Applications ‐Integration ‐Human dimension ‐Caring ‐Learning how to learn	Study: descriptive. Describes the experiences of two teachers. Objectives: Describing the meaning of ‘meaningful learning’, identifying principles of effective course design and providing two examples of idea implementation.	‐Lasting change ‐True understanding	Using the taxonomy of Fink to design courses that develop meaningful learning. Definition of objectives: 1. Understand and remember key concepts, terms, relationships, etc.; 2. Know how to use the content; 3. Be able to relate this issue with other issues; 4. Identify the personal and social implications of knowledge on this topic; 5. The value of this argument, as well as the value of further learning on this topic; 6. Know how to continue to learn about this topic, after the end of the course. Learning activities: use of active learning. Educational evaluation.
6‐Marks and McIntosh (2006)	Health Information Management students – University of Sydney's	Meaningful learning can be summarized as a continuous process based on permanent knowledge acquired and applied. The essence of significant learning is experiential learning. Learning from experience was considered according to Kolb's learning experience.	Study: descriptive. Objectives: This article shows how, through the process of experiential learning, professional experience can promote reflective thinking and meaningful learning, the ability to integrate theory and practice, as well as professional and personal development.	‐Learning through experience and critical reflection.	Internships at health facilities.
7‐Magnussen (2008)	Hawaii University in Manoa‐ School of Nursing and Dental Hygiene (SONDH)	Constructivist principles of learning, which allow students to build new knowledge based on their previous wealth of information. Fink's Taxonomy: he proposed that learning promotes change and lasting learning (i.e., meaningful learning that comes from understanding). This model is based on several specific types of learning, which can be identified in a taxonomy.	Study: descriptive. Describes the experience of a teacher. Objectives: application of the principles of meaningful learning environments e‐learning (implementation of a program‐online).	‐Construction of the person.	This article uses the experience of a teacher involved in a new online program to demonstrate the application of Fink's taxonomy. Method: ‐They have switched their curricula to the online process; ‐They have provided support to teachers; ‐Use of Fink's taxonomy for planning the course, formulation of objectives and learning assessment criteria; interviews with teachers for evaluation purposes.
8‐Lickteig (2004)	Scholl of nursing Georgia Southern University Statesboro Ga. Nursing students, Postgraduate Course in Mental Health.	Constructivism. Autobiographical inquiry.	Study: descriptive. Objective: Becoming familiar with various research designs, including the relevance of research for practice and appreciation for the knowledge provided by research.	‐Reflection on the experience: autobiography	Groups of 8–10 students were invited to reflect on their life experience, with regard to the identification of problems and formulation of hypotheses. They were encouraged to pinpoint some factors that could improve their lives. Students: – chose an interesting topic – researched the subject using any method, – presented and discussed in small groups the results in the class – time 20 minutes. They were urged to explore new areas of knowledge and how they could be used for professional practice.
9‐Tryssenaar and Gray (2004)	Scope: long‐term care staff in Thunder Bay, Ontario, Canada Involved professions: nurses and support staff.	Knowles (1990) suggests that relevance is the most important learning factor for an adult who invests their energies to understand what is important for troubleshooting. For the training to be meaningful, it must be relevant and brief because the operators only have a short period of time at their disposal and it must involve all staff.	Study: Descriptive Objective: Introducing an innovative programme of continuing education to all staff of the long‐term care facilities in Thunder Bay, Ontario, using different techniques ranging from lessons to narrative.	‐Content relevant to the needs	30‐minute sessions were tested including lessons and narrative based on the learners’ needs. The topics were identified through the use of PBL. Each session developed from one to three key concepts or ‘pearls of knowledge’ and took place in the afternoon change of shift. The sessions were repeated three times to allow the participation of all staff. The session was divided into five phases: ‐Welcoming of the participants fifteen minutes before the session to get to know each other ‐Theoretical component: presentation of the chosen topic ‐Affective component: using a story ‐Implementation: brief discussion and knowledge implementation ‐Relaxing after the formal part of the session
10‐Akinsanya and Williams (2004)	Diploma of Higher Education in Nursing and BSc Nursing courses UK	Second Concept maps Novak and Gowin. A conceptual map can be seen as a device for the schematic representation of a set of concepts and meanings. This is a strategy used by many tutors and facilitators to promote meaningful and deep learning.	Study: Descriptive Objectives: Introducing concept mapping (use of mind maps) as a tool for learning, teaching and assessment in a form of Inquiry‐Based‐Learning (IBL) in a high school nursing education.	‐Change. ‐Deep learning.	Students were divided into small groups as indicated by IBL and guidelines were provided and discussed for the construction of mental maps. They selected a topic of their interest, sought additional information and prepared a poster to be presented and discussed in the group of students. Finally, they implemented a self‐assessment of the work done and then received feedback from the facilitators.
11‐Irvine (1995)		Reference to Ausubel and Novak. Meaningful learning is based on the theory of assimilation by Ausubel *et al*. (1978).	Study: Narrative review Objectives: Exploring the nature of a concept map, the research on concept mapping and meaningful learning, possible reasons why its use has not developed in the training of nurses. By addressing these issues, the author hopes that educators of nurses will be aware of the use of concept maps and will promote meaningful learning.	‐Integration of new and acquired knowledge.	Using concept maps to foster meaningful learning.

Studies described in the selected papers were performed in Canada (1), Sweden (1), the USA (5), the UK (1), Australia (1) and Brazil (1), respectively, and the narrative review was by British authors. All articles but one were in English, the exception being in Portuguese. Several papers defined meaningful learning through a conceptual reference model or consulted bibliography and references influenced developmental and implementation methods chosen for each study.

After analysing the definitions, three main categories of characteristics of meaningful learning emerged:

- Meaningful learning as ‘active building‐up process’;
- Meaningful learning as ‘change’;
- Meaningful learning as ‘developed through experience’.

### Meaningful learning as ‘active building‐up process’

According to Johannsen et al. (2012), meaningful learning is an active process whereby newly acquired knowledge is interpreted against past knowledge, thereby fostering greater and more in‐depth understanding (Johannsen *et al*. 2012). The scholarship model deployed by the author is meant to help learners develop meaningful learning and self‐efficacy by encouraging them to relate already‐acquired knowledge to newly acquired information, put theory into practice and structure content to be mastered into a logical, coherent meaning continuum (Johannsen *et al*. 2012).

According to Johannsen (2012), meaningful learning coincides with deep learning which, in turn, contrasts with surface learning and identifies such issues as pertinence of current or future profession, or interest and authenticity of undertaken tasks – all of which are significant pegs onto which students may hitch meaningful learning (Johannsen *et al*. 2012). A further issue relating to education and knowledge transfer is the use of competence in new situations, viewed in terms of self‐efficacy – a field where learners may be in need. Self‐efficacy is defined as the belief that one is capable of performing in a certain manner to attain certain skills. Lowered perceived self‐efficacy limits the development of competence, thereby hindering education and preventing the development of meaningful learning (Johannsen *et al*. 2012).

According to Dreifuerst (2012), meaningful learning goes beyond memorizing information by promoting conceptual understanding and supporting the development of clinical reasoning skills as a guide to professional practice. Indeed, the author identifies debriefing, viewed as a strategy conducive to the development of clinical reasoning, as an effective way of triggering meaningful learning (Dreifuerst 2012). Used with nursing students, Debriefing in Meaningful Learning (DML) is a constructivist practice deploying guided reflective thinking to foster critical reasoning and thinking. This reflective method encourages learners’ to apply thought processes to clinical experience, thereby enhancing their knowledge, decision‐making skills and learning (Dreifuerst 2012). Coherence and cohesion between students’ positive perception of the learning environment and practical proof of acquired learning are key to best teaching practices and stances of meaningful learning experience in learners (Dreifuerst 2012).

According to Magnussen's (2008) constructivist view, individuals build their own learning. This clearly clashes against the traditional view of learning wherein information is handed down passively from expert to learner. If learning is to improve understanding and theory is to become practice, then both learners and professionals need to be equipped with relevant, meaningful competences (Magnussen 2008). Magnussen describes the experience of a group of instructors delivering online education through applying the principles of meaningful learning according to Fink's Taxonomy (2007) (Fink 2007, Magnussen 2008). Despite longer course‐planning times, instructors reported on greater success in reaching set educational goals and learners took an active part in their own learning which was, therefore, driven by personal needs (Magnussen 2008).

Irvine (1995) posits that meaningful learning entails a process whereby newly acquired and already‐mastered information interact (Irvine 1995) and authors of this review attempt to raise teaching practitioners’ awareness to the relevant use of concept mapping in meaningful learning. Concept maps are meta‐cognitive tools that help students organize their thoughts on and around a specific, or any, subject matter into a diagrammatic form. Over time, this tool has become key to developing meaningful learning in students, to curriculum planning, assessment, as well as to research. Moreover, the deployment of concept maps has enhanced instructors’ efficacy and improved students’ results. This topic is a matter of current discussion, as shown by the literature wherein an abundance of references can be found to experience with using concept mapping to foster meaningful learning and critical thinking skills (Daley & Torre 2010).

### Meaningful learning is ‘change’

According to Krueger *et al*. (2011), meaningful learning is an act, process or experience leading to the acquisition of knowledge or competences. Learning only takes places if it brings about change in the learner and only becomes meaningful if the change is long‐lasting (Krueger *et al*. 2011). Kreuger chose to re‐design a health policy training course through using Fink's Taxonomy (2007) in an attempt at encouraging significant learning, enhancing the efficacy of course teaching and fostering learner interest (Fink 2007, Krueger *et al*. 2011).

Prado *et al*. (2011) maintain that three conditions must co‐occur for meaningful learning to take place, i.e. learners must be willing to learn, hold some degree of relevant pre‐cognizance as well as logical and psychological meaning‐content to attribute to what they are about to acquire (Prado *et al*. 2011). The authors used Moodle to develop and evaluate a virtual classroom on the Theory of Meaningful Learning. Findings revealed that a virtual environment favours learning, both individually based and tutor‐mediated, thereby encouraging students to take on an active learning role. Prado and De Almeida believe that meaningful learning is indeed important in nursing education (Prado *et al*. 2011).

Fink (2007) agrees with the idea of learning as consolidated behavioural change, i.e. meaningful in the presence of true understanding. This author also maintains that learning becomes meaningful when newly acquired information is pegged onto a pre‐existing cognitive framework. Moreover, Fink posits that meaningful learning somehow comprises the following six learning types:

- Foundational knowledge: understanding and remembering information and ideas also underpin other learning types;
- Application: developing the ability to apply acquired skills (e.g. project management skills), meant to trigger critical, creative and/or practical thinking and foster other learning types;
- Integration: linking concepts, ideas, people and experience supplies professionals with new skills of an intellectual nature;
- Human dimension: learning to know oneself and others encourages professionals to relate to the human aspects of what they are acquiring;
- Caring: exploring new feelings, interests and values inherent in newly acquired learning helps to stimulate and involve professionals;
- Learning how to learn: the development of self‐driven learning skills allows professionals to carry on learning even once the experience in hand has been completed (Fink 2007, Magnussen 2008).

Meaningful learning is deemed paramount in nursing education, especially for students who are likely to become future nursing instructors, as they will be in the position to feed newly acquired knowledge into their professional performance and practice (Magnussen 2008).

Akinsanya and Williams (2004) agree that meaningful learning is characterized by change and, therefore, exhort teaching practitioners and tutors in nursing to use such strategies as concept mapping to promote and develop deep learning (Akinsanya & Williams 2004). In turn, learners are exhorted to look into the connections between concepts to develop greater understanding. In particular, these authors deploy concept maps to assess several nursing input modules and declare them to be ideal tools when attempting to highlight change.

### Meaningful learning as ‘developed through experience’

Marks and McIntosh (2006) maintain that learning is mediated by experience that allows for and facilitates deep, meaningful learning. Experience‐mediated learning correlates with deep learning style expressed in terms of understanding the meaning of acquired knowledge. Critical reflection is part and parcel of deep learning and paramount in experience‐based learning (Marks & McIntosh 2006). Indeed, Marks and McIntosh value experience‐based learning viewed in terms of training, as it contributes to the development of reflective thinking alongside meaningful learning by integrating theory and practice. Personal development is strongly associated with deep learning and critical reflection promotes professional development, thereby becoming an integral part of any permanent learning process.

Lickteig (2004) agrees with the assumption that learning occurs through reflecting on life experience from a personal, autobiographical perspective, as knowledge comes from individual experience. Far from being limited to recounting personal experience, autobiographical investigation addresses acquisition from the lookout of a piece of personal experience able to trigger new meaningful learning (Lickteig 2004). The author accordingly added an autobiographical component to her course curriculum on mental healthcare with the aim of fostering meaningful learning in nursing students, who later supplied positive feedback on the course itself.

Finally, Tryssenaar and Gray (2004) stated that meaningful educational content should meet learners’ needs and be as concise as possible, as professionals usually have limited time available for learning. Again, these same authors believe that to foster sharing, meaningful content should address all professionals (Tryssenaar & Gray 2004). Tryssenaar and Gray consequently fed these principles into an innovative curriculum for ongoing education, featuring short sessions, Problem‐Based Learning (PBL), narrative and practice in applying newly acquired knowledge, which appears to hold greater efficacy than traditionally deployed pedagogical methods.

## Discussion

The literature reports several learning types, offering relevant definitions and underlying developmental methodology and implementation tools. Focused as it is on identifying the characteristics of meaningful learning according to the constructivist paradigm, this review has singled out three main features: (1) meaningful learning as ‘active building‐up process’; (2) meaningful learning as change; (3) meaningful learning as ‘experience‐mediated knowledge’.

In addition, we found that the literature mentions ‘reflective learning’ (Liimatainen *et al*. 2001), ‘transformative learning’ (Parker & Myrich 2010), ‘vicarious learning’ (Roberts 2010) and ‘generative learning’ (Jonassen 1999), although similarities and/or differences between these are somewhat unclear and can generate confusion. Therefore, further research would be needed to clarify and describe these concepts.

One of the main difficulties encountered by us was, however, the apparent overlap in use by the literature of the terms meaningful and significant. In fact, most sources used the expression ‘Meaningful Learning’ (Irvine 1995, Akinsanya & Williams 2004, Lickteig 2004, Tryssenaar & Gray 2004, Marks & McIntosh 2006, Dreifuerst 2012, Johannsen *et al*. 2012) and only in four did the authors use the expression ‘Significant Learning’ (Fink 2007, Magnussen 2008, Krueger *et al*. 2011, Prado *et al*. 2011). A preliminary scan of the selected sources led us to believe that the above expressions were used synonymously; however, a closer investigation revealed that ‘Meaningful Learning’ actually defines a cognitive and meta‐cognitive level of processing (Novak 2001), whereas ‘Significant Learning’ also encompasses behavioural and affective levels (Fink 2007).

While investigating, we encountered the novel term ‘Deep Learning’, which was used to define in‐depth, long‐lasting learning leading to wider, deeper comprehension (Akinsanya & Williams 2004, Johannsen *et al*. 2012). ‘Deep Learning’, intended as learning style, has been investigated and measured by several researchers (Bigg 2001, Snelgrove 2004).

The literature reporting on deep learning experience and its relevant underlying strategies, as yet, is limited. Exposure to traditional or mechanical pedagogy signifies that conservative teaching methodologies are still fairly popular among pedagogues and professionals, who tend to draw from and re‐enact such teaching patterns in their own teaching and learning development sessions. Quite often, it is the teaching practitioners who perform the mental work expected of learners (Irvine 1995).

### Limitations

For this study, only papers referring to the constructivist paradigm were selected and this reduced the number of articles included in the review. We did not set any time restrictions, so also out‐dated papers were retrieved.

## Conclusion

Besides being informed by active and innovative models and tools, constructivism, clinical thinking and ethical comportment (Benner *et al*. 2010), education for healthcare professionals is science‐ and evidence‐based. In health care, education aims at preparing highly competent and skilled professionals geared for quality changes, the latter being deemed a must when operating in complex environments (Watson 2008). In addition, education aims at facilitating the development of meaningful learning and at fostering interdependence among professionals (Frenk *et al*. 2010). However, the complexity and results of learning, teaching and assessment strategies of inputs given to healthcare professionals and nurses certainly constitute a problem (Akinsanya & Williams 2004).

Several constantly interacting factors, such as personality and learning approach, contribute to and influence learning. Personal characteristics include such aspects as pre‐existing knowledge, both theoretical and practical; learning skills and style, learning context and the need to develop diverse strategies able to provide chances for meaningful learning to occur (Tryssenaar & Gray 2004, Johannsen *et al*. 2012).

Inclusion of all categories of healthcare professionals, rather than limiting research to nurses (most frequently studied) enabled to highlight the subject matter of deep learning and its relevant underlying methodology and strategies. Moreover, the constructivist paradigm helped to define a new outlook on learners, who are currently becoming increasingly important in and accountable for deep, ongoing, durable learning. Strategies able to influence and drive pedagogical practice among healthcare professionals towards meaningful learning require further investigation. Future research findings may yield useful insights into similarities and differences that might help address learner needs more adequately; explore innovative pedagogical strains and trains of thought in nursing and healthcare education; revisit clinical training with a view to devising new practice‐mediated learning methods; perform pedagogical and andragogical research and ultimately support the efficacy and significance of already‐undertaken innovations.

## Conflict of interest

The Authors declare that there is no conflict of interest.

They also declare that they agree with the content of this manuscript, which has not been published or submitted for publication elsewhere.

## Author contributions

All authors have agreed on the final version and meet at least one of the following criteria [recommended by the ICMJE (http://www.icmje.org/ethical_1author.html)]:

- substantial contributions to conception and design, acquisition of data, or analysis and interpretation of data;
- drafting the article or revising it critically for important intellectual content.
